# Metadherin facilitates podocyte apoptosis in diabetic nephropathy

**DOI:** 10.1038/cddis.2016.335

**Published:** 2016-11-24

**Authors:** Wen-Ting Liu, Fen-Fen Peng, Hong-Yu Li, Xiao-Wen Chen, Wang-Qiu Gong, Wen-Jing Chen, Yi-Hua Chen, Pei-Lin Li, Shu-Ting Li, Zhao-Zhong Xu, Hai-Bo Long

**Affiliations:** 1 Department of Nephrology, ZhuJiang Hospital, Southern Medical University, Guangzhou 510280, China; 2 Department of Nephrology, NanFang Hospital, Southern Medical University, Guangzhou 510515, China; 3 Department of Emergency, ZhuJiang Hospital, Southern Medical University, Guangzhou 510280, China

## Abstract

Apoptosis, one of the major causes of podocyte loss, has been reported to have a vital role in diabetic nephropathy (DN) pathogenesis, and understanding the mechanisms underlying the regulation of podocyte apoptosis is crucial. Metadherin (*MTDH*) is an important oncogene, which is overexpressed in most cancers and responsible for apoptosis, metastasis, and poor patient survival. Here we show that the expression levels of Mtdh and phosphorylated p38 mitogen-activated protein kinase (MAPK) are significantly increased, whereas those of the microRNA-30 family members (miR-30s) are considerably reduced in the glomeruli of DN rat model and in high glucose (HG)-induced conditionally immortalized mouse podocytes (MPC5). These levels are positively correlated with podocyte apoptosis rate. The inhibition of Mtdh expression, using small interfering RNA, but not Mtdh overexpression, was shown to inhibit HG-induced MPC5 apoptosis and p38 MAPK pathway, and Bax and cleaved caspase 3 expression. This was shown to be similar to the effects of p38 MAPK inhibitor (SB203580). Furthermore, luciferase assay results demonstrated that Mtdh represents the target of miR-30s. Transient transfection experiments, using miR-30 microRNA (miRNA) inhibitors, led to the increase in Mtdh expression and induced the apoptosis of MPC5, whereas the treatment with miR-30 miRNA mimics led to the reduction in Mtdh expression and apoptosis of HG-induced MPC5 cells in comparison with their respective controls. Our results demonstrate that Mtdh is a potent modulator of podocyte apoptosis, and that it represents the target of miR-30 miRNAs, facilitating podocyte apoptosis through the activation of HG-induced p38 MAPK-dependent pathway.

Podocyte loss is emerging as a central pathological mechanism underlying diabetic nephropathy (DN). Podocyte apoptosis is one of the major causes of podocyte loss^1^ and one of the earliest cellular lesions affecting the diabetic kidney.^2^ Recent studies revealed that many pathways are involved in this process, including Notch,^3^ transforming growth factor *β* (TGF-*β*),^4^ and p38 mitogen-activated protein kinase (MAPK)^2, 5^ signaling pathways. However, cellular and molecular pathomechanisms responsible for podocyte apoptosis in DN remain largely unknown.

Here we evaluated the involvement of metadherin (Mtdh, also known as astrocyte elevated gene-1 (AEG-1)^6^ or lysine-rich CEACAM1 co-isolated (LYRIC)^7^) in this process. A comprehensive and convincing body of data highlights *MTDH* as an important oncogene that is overexpressed in all previously investigated cancers.^8^ It is involved in cancer cell proliferation, survival, autophagy, migration, invasion, apoptosis, angiogenesis, metastasis, and treatment resistance.^9^ In addition to its role in tumorigenesis, MTDH is a pleiotropic protein involved in various physiological and pathological processes, including development,^10^ neurodegeneration,^11^ inflammation,^12, 13, 14^ and epithelial–mesenchymal transition (EMT).^15^ MTDH has a role in these processes through the regulation of various signaling pathways, including transcription factor nuclear factor-κB (NF-κB), GTPase HRAS, phosphoinositide 3-kinase (PI3K)/AKT, p38 MAPK, and WNT pathways.^15, 16^ Recently, Eisenrech *et al.*^17^ demonstrated that podoplanin, a factor involved in cancer biology but poorly characterized and functionally analyzed in podocytes, is antiapopotic in AngII-induced human podocytes, and regulated by miR-29b. However, the expression and role of MTDH in podocytes have not been investigated before.

p38 MAPK is one of the important member of MAPKs, activated by proinflammatory cytokines or cellular stress, such as genotoxic, osmotic, hypoxic, or oxidative stress.^18^ Previous studies have implicated p38 MAPK in a variety of cellular activities, including cell survival, death, proliferation, differentiation, and transformation.^19^ It was shown to regulate apoptosis by modulating the activities of the Bcl-2 family proteins.^20^ Furthermore, P38 MAPK was implicated in podocyte apoptosis induced by doxorubicin (Adriamycin)^21^ and high serum lipopolysaccharide levels.^22^

Data obtained over the past decade have demonstrated that microRNAs (miRNAs) have vital roles in the cell growth, proliferation, differentiation, apoptosis, and stress response.^23^ Extensive investigations showed that MTDH represents a target gene of several miRNAs.^16^ miR-30 family consists of five members, miR-30a–miR-30e, and it was shown that they are involved in hepatocyte EMT,^24^ cancer cell apoptosis and autophagy,^25^ and cardiomyocyte apoptosis.^26^ Moreover, Li *et al.*^27^ reported that the overexpression of miR-30a-5p inhibits cell proliferation, colony formation, and induces apoptosis in liver cancer cells by targeting MTDH. Members of miR-30 family were shown to be abundantly expressed in podocytes, and the downregulation of their expression facilitates podocyte apoptosis in glomerulosclerosis.^28^

In this study, we investigated the potential involvement of Mtdh in podocyte apoptosis, together with the interactions between the members of miR-30 family and the changes in Mtdh expression. We demonstrate here that MAPK signaling is required for Mtdh-mediated induction of podocyte apoptosis.

## Results

### Apoptosis rate is increased DN and HG-induced MPC5 cells

**Figure 1:**
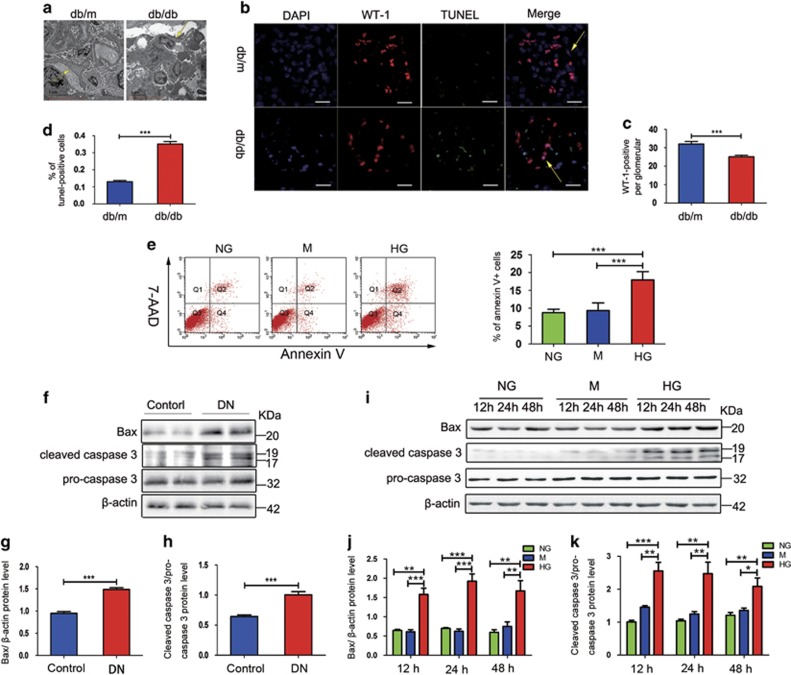
Apoptosis rate is increased DN and HG-induced MPC5 cells. (**a**) Thickening of glomerular basement membrane, and the shortening and effacement of podocyte foot process were observed in db/db mice (yellow arrows). Scale bars, 5 *μ*m. (**b**) Representative images showing WT-1 and TUNEL double staining. Scale bars, 20 *μ*m. (**c**) Quantification of WT-1-positive cells in glomerular sections. The number of WT-1-positive cells was obtained from three randomly selected glomeruli per kidney section (*n*=9 per group). (**d**) Quantification of the podocyte apoptosis rate in kidney glomeruli of db/m and db/db mice. Values are presented as the percent of double-labeled/WT-1-labeled cells from three randomly selected glomeruli per kidney section (*n*=9). (**e**) Apoptosis rate of MPC5 cells treated with NG, M, and HG for 48 h (*n*=4). (**f**) Immunoblotting for Bax and cleaved caspase 3 expression in glomeruli from streptozotocin-induced diabetic rats and the controls. (**g–h**) Quantification of Bax and cleaved caspase 3 expression *in vivo* (*n*=6). (**i**) Bax and cleaved caspase 3 expression in MPC5 cells stimulated with NG, M, and HG for 12, 24, or 48 h. (**j–k**) Quantification of the results presented in (**l**) (*n*=3). Bars represent mean±S.E.M. *P<0.05, **P<0.01, and ***P<0.001

The results obtained using electron microscopy showed the evidence of glomerular basement membrane thickening, effacement, and focal fusion of the podocyte foot process in db/db mice (Figure 1a).

We evaluated podocyte apoptosis in db/db and db/m mice by terminal deoxynucleotidyl transferase dUTP nick end labeling (TUNEL) staining combined with WT-1 (podocyte nucleus marker) immunofluorescent staining. WT-1 immunostaining showed a significant reduction in the number of podocyte in the glomeruli of db/db mice (Figures 1b and c). In contrast, a significant increase in the intensity of TUNEL staining of the podocytes in db/db mice was observed in comparison with db/m (Figures 1b and d). We next estimated the apoptosis rate of MPC5 cells treated with 5.3 mM glucose (NG, normal glucose), 5.3 mM glucose+25 mM mannitol (M, an osmotic control), or 30 mM d-glucose (HG, high glucose) for 48 h. Consistent with the TUNEL staining, flow cytometry analysis showed that the rate of apoptosis was significantly increased in HG-treated group compared that in control groups (Figure 1e). We then isolated the glomeruli from streptozotocin-induced diabetic rats for western blot analysis (Supplementary Figure 1). Result shown that the expression levels of Bax and cleaved caspase 3, two known indicators of apoptosis, were significantly increased in the obtained from streptozotocin-induced diabetic rats (Figures 1f–h), as well as in HG-induced MPC5 cells (Figures 1i–k), in comparison those in their respective controls.

### Mtdh expression is enhanced *in vivo* and *in vitro* models of DN

**Figure 2:**
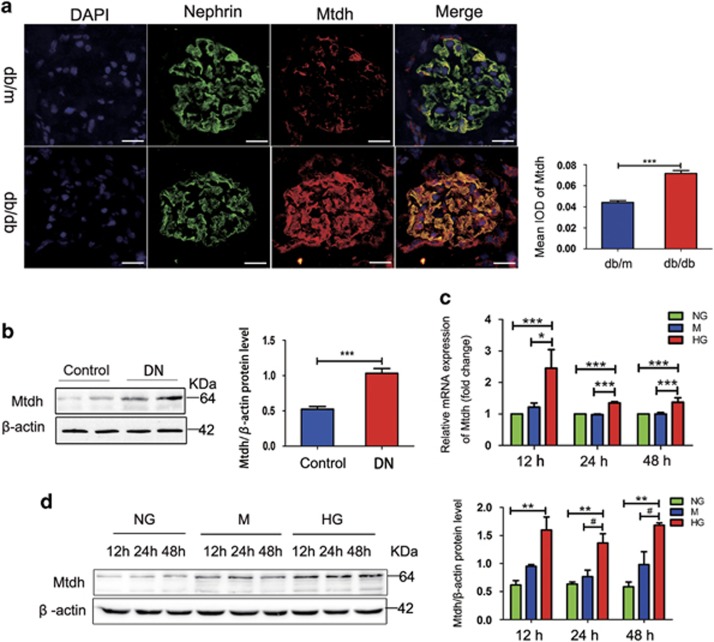
Mtdh expression is increased in *vivo* and *in vitro* models of DN. (**a**) Nephrin and Mtdh double staining. Scale bars, 20 *μ*m. (**b**) Mtdh protein expression in the glomeruli of DN rats (*n*=6). (**c**) Mtdh expression levels in MPC5 cells treated with NG, M, or HG for 48 h (*n*=3). (**d**) Mtdh protein expression levels in MPC5 cells treated with NG, MA, or HG for 12, 24, or 48 h (*n*=3). Bars represent mean±S.E.M. *P<0.05, **P<0.01, ***P<0.001 and ^#^*P*<0.05

To determine whether Mtdh is expressed in podocytes, we performed double fluorescence staining of nephrin (podocyte marker) and Mtdh. Our results demonstrated that Mtdh is expressed not only in the tubules but also in the glomeruli of the investigated mice. Furthermore, the colocalization of Mtdh and nephrin indicated that Mtdh is predominantly expressed in glomerular podocytes. The intensity of Mtdh staining was shown to be much stronger in db/db mice than in db/m mice (Figure 2a). Western blot analyses demonstrated that Mtdh expression is significantly elevated in the DN glomeruli in comparison with that in the control (Figure 2b). In addition, western blot analyses demonstrated that, following the treatment of MPC5 cells with HG (from 0 to 50 mM range), the expression of Mtdh considerably increased (Supplementary Figure 2). Furthermore, Mtdh mRNA expression was significantly increased in HG-induced MPC5 cells in comparison with the control (Figure 2c). This was accompanied by a significant increase in the protein levels of Mtdh in HG-induced MPC5 cells at different time points (12, 24, or 48 h), although not in a time-dependent manner (Figure 2d).

### Mtdh knockdown suppresses HG-induced MPC5 apoptosis, but Mtdh overexpression promotes apoptosis

**Figure 3:**
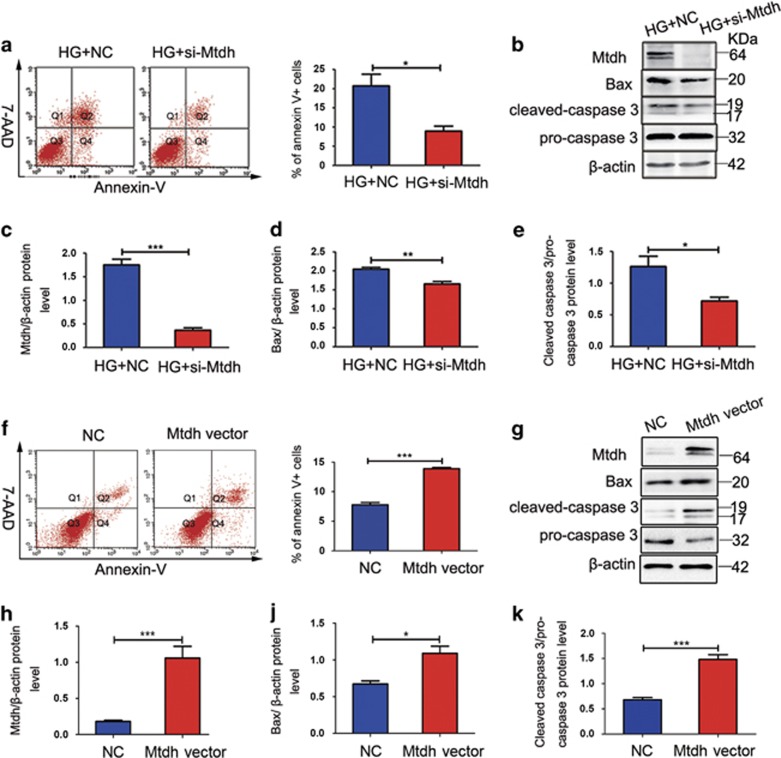
Mtdh expression levels affect the apoptosis of HG-induced MPC5 cells. (**a**) Flow cytometry analysis of the apoptosis rate of MPC5 cells transfected with NC or si-Mtdh and treated with HG for 48 h (*n*=3). (**b**) Mtdh, Bax, and cleaved caspase 3 protein expression levels in MPC5 cells following the transfection with NC or si-Mtdh and stimulation with HG for 48 h. (**c–e**) Quantification of Mtdh (**c**), Bax (**d**), and cleaved caspase 3 (**e**) expression in MPC5 cells (*n*=3). (**f**) Flow cytometry analysis of the apoptosis rate of MPC5 cells transfected with control vector (CV) or Mtdh overexpression vector (*n*=3). (**g**) Mtdh, Bax, and cleaved caspase 3 protein expression levels in MPC5 cells following the transfection with CV or Mtdh overexpression vector. (**h–k**) Quantification of Mtdh (**h**), Bax (**j**), and cleaved caspase 3 (**k**) expression in MPC5 cells (*n*=3 each). Bars represent mean±S.E.M. *P<0.05, **P<0.01, and ***P<0.001

We further investigated the role of Mtdh in the induction of apoptosis by *Mtdh* knockdown or overexpression. The results obtained using flow cytometry showed that the rate of apoptosis of HG-induced MPC5 cells transfected with small interfering RNAs (siRNAs) targeting Mtdh (si-Mtdh) considerably decreased compared with the negative control (NC) group (Figure 3a). Successful Mtdh knockdown was confirmed by western blot analysis. Mtdh expression in HG-induced MPC5 cells was shown to be considerably decreased following the knockdown (Figures 3b and c). In addition, the inhibition of Mtdh expression was shown to suppress the expression of Bax and cleaved caspase 3 in HG-induced MPC5 cells (Figures 3b, d, and e).

Following the overexpression of Mtdh in MPC5 cells, we showed that the apoptosis rate was significantly elevated in the Mtdh overexpression group compared with control vector-treated cells (Figure 3f). Similarly, Mtdh overexpression led to an increase in both Bax and cleaved caspase 3 levels in MPC5 (Figures 3g–k).

### Mtdh regulates MPC5 apoptosis through the p38 MAPK pathway

**Figure 4:**
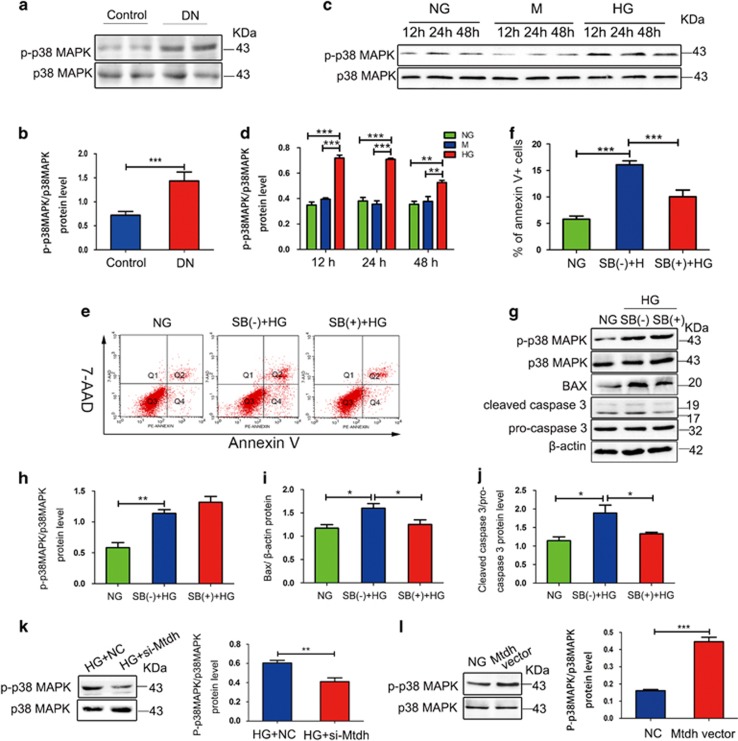
Mtdh facilitates MPC5 apoptosis through the activation of p38 MAPK pathway. (**a**) p-p38 MAPK expression in the samples obtained from the glomeruli of DN and control rat. (**b**) Quantification of the results presented in **a** (*n*=6). (**c**) p-p38 MAPK expression in HG-induced MPC5 cells for 12, 24, and 48 h. (**d**) Quantification of the results presented in **c** (*n*=3). (**e**) Apoptosis of MPC5 cells treated with NG, HG, and HG, and p38 MAPK inhibitor (p38I) for 48 h. (**f**) Quantification of the results presented in **e** (*n*=3). (**g**) p-p38, Bax, and cleaved caspase 3 expression levels in MPC5 cells treated with NG, HG, and HG, and p38I for 48 h. (**h–j**) Quantification of p-p38 MAPK (**h**), Bax (**i**), and cleaved caspase 3 (**j**) expressions (*n*=3). (**k**) p-p38 MAPK expression levels in NC or si-Mtdh-treated MPC5 incubated with HG for 48 h (*n*=3). (**l**) p-p38 MAPK expression levels in control vector or Mtdh overexpression vector-treated cells (*n*=3). Bars represent mean±S.E.M. *P<0.05, **P<0.01, and ***P<0.001

To investigate whether Mtdh-induced apoptosis of HG-induced MPC5 cells is mediated by the p38 MAPK pathway, we measured the expression of phosphorylated (p)-p38 MAPK in the glomeruli from DN rats and HG-induced MPC5 cells. The expression levels of p-p38 MAPK were shown to be significantly increased in the DN glomeruli (Figures 4a and b) and HG-induced MPC5 cells (Figures 4c and d), in comparison with their respective controls. In addition, we showed that p38 MAPK pathway activation is involved in HG-induced MPC5 apoptosis, using p38 MAPK inhibitor, SB203580. Flow cytometry analysis showed that SB203580 treatment significantly inhibits HG-induced apoptosis in MPC5 cells (Figures 4e and f). Furthermore, the suppression of p38 MAPK pathway activation by this inhibitor led to a decrease in Bax and cleaved caspase 3 expression levels in comparison with the control, although the basal levels of p-p38 did not change (Figures 4g–j). MPC5 cells were then pretreated with si-Mtdh or NC, and treated with HG for 48 h. As demonstrated, si-Mtdh treatment led to a decrease in p-p38 MAPK expression in comparison with the NC group (Figure 4k), and Mtdh overexpression was shown to increase the expression of p-p38 MAPK in comparison with the control group (Figure 4l).

### Mtdh represents a direct target of the members of miR-30 family

**Figure 5:**
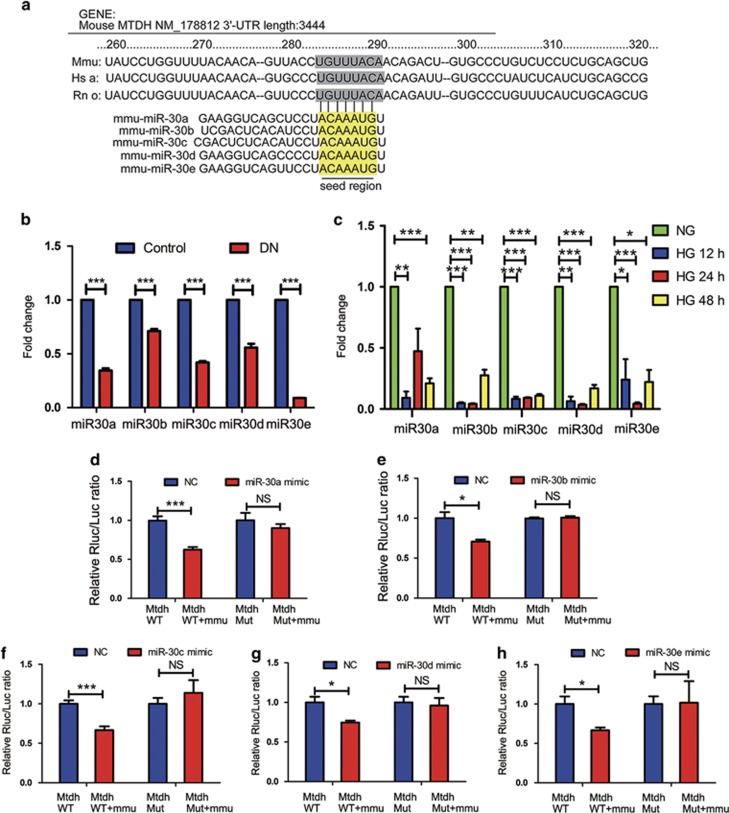
Mtdh represents a direct target of the members of miR-30 family. (**a**) Predicted target sequence of miR-30s in Mtdh 3′-UTR in different species and the mouse 3′-UTR was study in our experiment. (**b** and **c**) The expression of miR-30s in the glomeruli of DN or control (**b**) and HG-induced MPC5 cells in different time points (0, 12, 24, and 48 h) (**c**). U6 was used for the normalization of the obtained levels (*n*=3). (**d–h**) Luciferase activity measured in MPC5 co-transfected with miR-30a, -30b, -30c, -30d, and -30e mimics, and NC, and the luciferase reporter for Mtdh wild-type (WT) or mutant (Mut) 3′-UTR (*n*=3). Bars represent mean±S.E.M. *P<0.05, **P<0.01, and ***P<0.001

The identification of putative Mtdh-regulating miRNAs, using TargetScan v5.1 (Cambridge, MA, USA), showed that 3′-untranslated region (UTR) of Mtdh contains seed sequences of miR-30s and that these sequences are highly conserved in diverse species (Figure 5a). Therefore, we studied miR-30 expression in the DN glomeruli and HG-induced MPC5 cells. As shown in Figure 5b, miR-30s were significantly downregulated in the DN glomeruli in comparison with the control. In addition, the levels of miR-30s were considerably decreased in MPC5 cells stimulated with HG for 12, 24, and 48 h (Figure 5c). These results suggest that miR-30s may have a role in DN development.

Next, luciferase reporters, containing normal or mutated binding sites for miR-30s, were constructed. The obtained results demonstrated that miR-30a, -30b, -30c, -30d, and -30e mimics can significantly inhibit the luciferase activity of the wild-type Mtdh 3′-UTR reporter, but not that of the NC, and that this inhibition was reduced when the mutant reporter, with mutated miR-30-binding site, was used (Figures 5d–h).

### miR-30s lead to the reduction in Mtdh expression in HG-induced MPC5 cells

**Figure 6:**
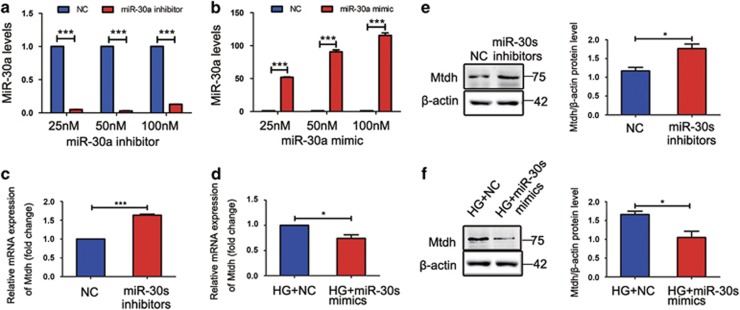
miR-30s inhibit Mtdh expression in HG-induced MPC5 cells. (**a**) miR-30a levels after the transient transfection of MPC5 cells with miR-30a inhibitor with different concentrations or their corresponding NCs (*n*=3). (**b**) miR-30a levels after the transient transfection of MPC5 cells with miR-30a mimic with different concentrations or their corresponding NCs (*n*=3). (**c**) Mtdh mRNA levels after the transient transfection of MPC5 cells with miR-30 inhibitors or their corresponding NCs (*n*=3). (**d**) Mtdh mRNA levels in MPC5 cells after the transient transfection with miR-30 mimics or the corresponding NCs, and the following 48 h treatment with HG (*n*=4). (**e**) Mtdh protein expression levels in MPC5 cells treated as in **c** (*n*=3). (**f**) Mtdh protein expression levels in MPC5 cells treated as in **d** (*n*=3). Bars represent mean±S.E.M. *P<0.05, **P<0.01, and ***P<0.001

To assess the effects of miR-30s on the expression of Mtdh, we transiently transfected MPC5 cells with miR-30 inhibitors, synthetic miRNA mimics, or their NCs. As shown in Figures 6a and b, transient transfection of MPC5 cells with miR-30a inhibitor resulted in significant reduction of miR-30a expression, and miR-30a mimic treatment considerably increased miR-30a levels. Mtdh mRNA level was shown to be significantly increased following the treatment with miR-30 inhibitors (Figure 6c), whereas miR-30s mimics led to a considerable reduction of Mtdh expression induced by HG (Figure 6d), compared with the corresponding NC treatment groups. Mtdh protein expression was shown to be increased as well after the treatment with the miR-30 inhibitors (Figure 6e), whereas miR-30 mimics significantly reduced Mtdh expression in HG-induced MPC5 cells (Figure 6f).

### miR-30s reduce the rate of HG-induced MPC5 apoptosis

**Figure 7:**
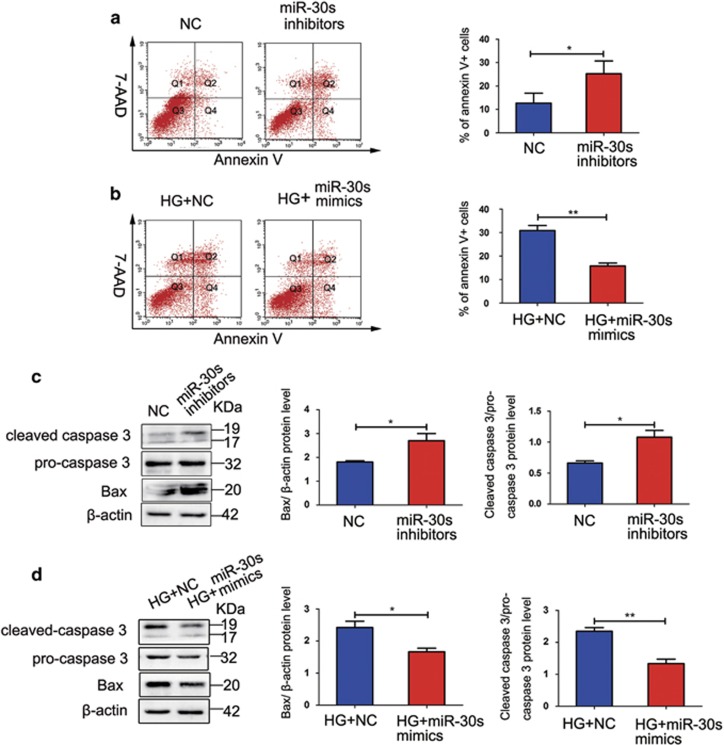
miR-30s reduce the rate of HG-induced MPC5 apoptosis. (**a**) miR-30 inhibitors significantly increase MPC5 apoptosis (*n*=3). (**b**) miR-30 mimics inhibit HG-induced MPC5 apoptosis (*n*=3). (**c**) miR-30 inhibitors induce the expression of Bax (*n*=3) and cleaved caspase 3 (*n*=4). (**d**) miR-30 mimics suppress the HG-induced upregulation of Bax and cleaved caspase 3 (*n*=3). Bars represent mean±S.E.M. *P<0.05, **P<0.01, and ***P<0.001

MPC5 were transfected with miR-30 inhibitors, mimics, or the respective NCs. Flow cytometry analysis demonstrated that the transfection of cells with miR-30 inhibitors significantly increased the percentage of apoptotic cells compared with the NC group (Figure 7a). Conversely, miR-30 mimics considerably reduced the rate of MPC5 apoptosis induced by HG (Figure 7b). Furthermore, miR-30 inhibitors significantly increased the expression of Bax and cleaved caspase 3 (Figure 7c). The treatment with miR-30 mimics considerably decreased HG-induced increase in the levels of these proteins (Figure 7d).

## Discussion

**Figure 8:**
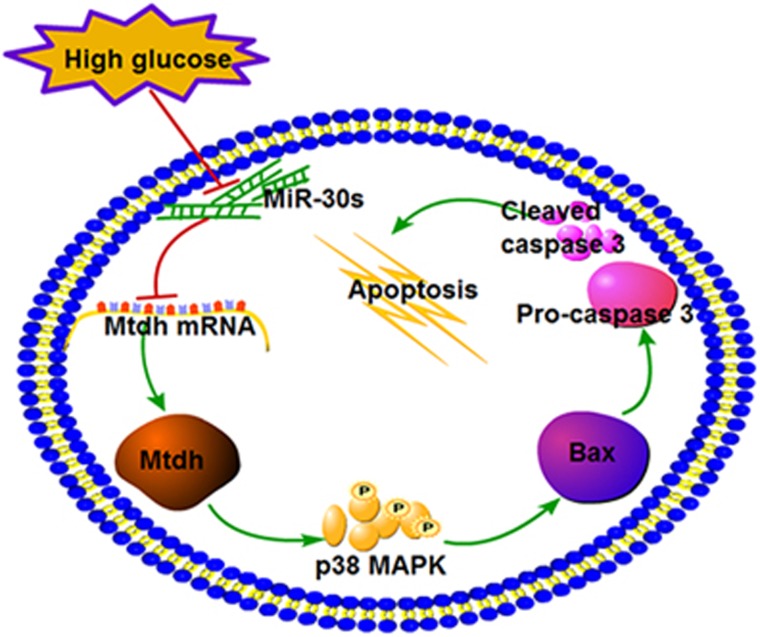
The proposed Mtdh-modulated apoptosis induction network in MPC5 cells miR-30s target Mtdh in HG-induced MPC5 cells. HG downregulates miR-30 family member expression and further increases Mtdh levels, which induces MPC5 apoptosis through the activation of p38 MAPK signaling pathway

Podocyte apoptosis is one of the major causes of podocyte loss and it has a crucial role in DN pathogenesis.^1^ Podocytes are exposed to various stimuli, such as hyperglycemia, fatty acids, growth factors, cytokines, and hormones, in diabetes mellitus. It has been demonstrated that, both *in vivo* and *in vitro*, HG levels lead to the activation of a series of event cascades in the kidneys, which may result in podocyte apoptosis. These may include increased oxidative stress, one of the most studied stimuli,^29, 30^ the activation of RAS, the induction of TGF-*β*, and the formation of advanced glycation end products.^31^ Langer *et al.*^32^ and Huang *et al.*^33^ demonstrated that HG (30 mM) induces podocyte apoptosis *in vitro*. Consistent with this, we showed that the apoptosis rate of podocytes is significantly increased in db/db mice, a well-recognized mouse model of type 2 diabetes, and that the increase in glucose levels is sufficient to induce apoptosis in MPC5. However, we suggest a different mechanism of HG-induced podocyte apoptosis, supported by the results presented in this study that showed that the downregulation of miR-30s leads to Mtdh upregulation, inducing MPC5 apoptosis mediated by the activation of p38 MAPK pathway (Figure 8).

Previously, MTDH expression was determined in glucose-induced proximal tubular epithelial cells.^34^ Recent studies have demonstrated that MTDH knockdown leads to the inhibition of cell growth and induces the apoptosis in human retinoblastoma as well as in hepatocellular carcinoma.^35, 36^ Here we showed that Mtdh is expressed in the tubules as well as the glomeruli, predominantly in podocytes. Furthermore, Mtdh expression was shown to be increased in the renal tissues of db/db mice, in the glomeruli form streptozotocin-induced diabetic rats and in HG-induced MPC5, and that Mtdh inhibition leads to the reduction in HG-induced MPC5 apoptosis. These findings indicate that Mtdh has a crucial role as a positive regulator of HG-induced apoptosis in these cells.

Recently, numerous studies demonstrated that MTDH is involved in many signaling pathways including NF-κB, PI3K/AKT, Wnt/*β*-catenin, MEK/ERK, and p38 MAPK pathways.^15, 16, 37^ In addition, the p38 MAPK pathway was shown to have an important role in podocyte injury.^38, 39^ The data obtained in this study showed that the expression of p-p38 is significantly enhanced in the DN glomeruli and HG-induced MPC5 cells, and it is accompanied by the increase in the expression of Bax and cleaved caspase 3. The inhibition of Mtdh led to the suppression of the activation of p38 MAPK pathway following the HG treatment, which suggests a possible link between Mtdh and p38 MAPK activation.

miRNAs were implicated in podocyte apoptosis as well. Eisenrech *et al.*^17^ demonstrated that miR-29b is involved in the regulation of apoptosis in podocytes through the regulation of podoplanin. Several miRNAs were found to regulate the expression of MTDH, and they may be involved in the pathogenesis of different diseases.^8^ These miRNAs include miR-26a, miR-203, miR-22, miR-375, and other.^16^ miR-30s were demonstrated to have a protective role in podocyte injury and to be involved in actin fiber disruption and podocyte apoptosis.^28^ Liu *et al.*^40^ suggested that MTDH can induce EMT in human non-small cell lung cancer through the regulation of miR-30a activity, whereas He *et al.*^41^ showed that miR-30a-5p suppresses the proliferation of hepatocellular carcinoma cells and enhances their apoptosis by targeting MTDH. A study by Li *et al.*^27^ showed that miR-30a-5p induced liver cancer cell apoptosis via the MTDH/PTEN/AKT pathway. We also found the activation of PTEN/AKT pathway in HG-induced MPC5 (Supplementary Figure 3). Here we confirmed that Mtdh represents a target of mi-R30s in DN, and that the expression of all five miR-30 family members is downregulated in the glomeruli form streptozotocin-induced diabetic rats and HG-induced MPC5 cells. In addition, our results agree with the results of a previous study,^28^ where miR-30s were found to protect podocytes from apoptosis induced by HG. However, the disagreement between the results of our study and the study conducted by He *et al.*^41^ and Li *et al.*^27^ may be due to the differences in the disease background and the alternative sources of the cells used in these studies.

In conclusion, we found that the upregulation of Mtdh facilitates podocyte apoptosis by inducing the activation of p38 MAPK signaling pathway. The obtained data indicate that miR-30s modulate Mtdh-induced podocyte apoptosis by targeting Mtdh 3′-UTR directly. Although the protective roles of miR-30s and Mtdh inhibition in DN have not been completely confirmed, we propose a novel cellular and molecular mechanism that may induce podocyte apoptosis in DN and provide new insights into the role of Mtdh.

## Materials and Methods

### Animal models

Diabetic db/db mice C57BL/KsJ (BKS.Cg-Dock7^m^+/+Lepr^db^/J) and their normal littermates (db/m; *n*=9 in each group) of both sexes, aged 6 weeks, were purchased from the Model Animal Research Center at Nanjing University, China. During the experiments, all animals were housed in standard animal facilities, at 22–26 °C and 40–70% humidity, and a 12/12 h light/dark cycle. All animal were killed at the age of 29 weeks. The study design and all animal procedures conformed to National Institutes of Health guidelines and were approved by the ethics committee for the experimental use of animals at Southern Medical University, Guangzhou, China (L2015046).

### Cell culture

Conditionally immortalized mouse podocytes (MPC5), between passages 12 and 20 (kindly provided by Professor Peter Mundel, Mount Sinai School of Medicine, USA, through Professor Wei Shi, Guangdong General Hospital, China), were cultured as previously described.^42^ In brief, undifferentiated podocytes were cultured in collagen I-coated dishes (BD Biosciences, Bedford, MA, USA) at 33 °C (permissive temperature) and 5% CO_2_ in RPMI 1640 (Gibco BRL, Gaithersburg, MD, USA) containing 10% fetal bovine serum (FBS; Gibco BRL), penicillin (100 U/ml), streptomycin (Gibco BRL; 100 mg/ml), and 50 IU/ml of recombinant murine IFN-*γ* (Peprotech, Rocky Hill, CT, USA). To induce differentiation, the cells were transferred to 37 °C (nonpermissive temperature) for 10–14 days and the medium was replaced with RPMI 1640 containing 5% FBS without IFN-*γ*. For the experiments, the medium was replaced by Dulbecco's modified Eagle medium (DMEM), containing 5.3 mM glucose (Invitrogen, Carlsbad, CA, USA) supplemented with 1% FBS. The differentiated podocytes were treated with 5.3 mM glucose (NG group), 5.3 mM glucose+25 mM mannitol (M group; Beijing Dingguo Changsheng Biotechnology, Beijing, China), 30 mM d-glucose (HG group; Sigma-Aldrich, St. Louis, MO, USA) for 12, 24, and 48 h, or HG plus SB203580 (Abcam, Cambridge, USA; 10 *μ*M) for 48 h, depending on the experiment.

### Apoptotic nucleus scoring *in situ*

To identify podocyte apoptosis *in vivo*, we performed double WT-1 and TUNEL^7^ staining. In brief, renal frozen sections were incubated with anti-WT-1 polyclonal antibody (1:50; Abcam) overnight at 4 °C. After the washing, a fluorescent secondary antibody, Alexa Fluor 546 (1:1000; Invitrogen) was applied for 6 h at 4 °C, and the samples were rinsed with phosphate-buffered saline (PBS; Wuhan Boster Biological Technology, Wuhan, China). Afterward, TUNEL staining was performed using the *In Situ* Cell Death Detection kit (Roche Molecular Biochemicals, Mannheim, Germany), according to the manufacturer's protocol. The specimens were counterstained with 4′,6-diamidino-2-phenylindole (DAPI; BestBio, Shanghai, China) for 10 min, and following this, the images were obtained with a light microscope (Ni-U, Nikon Corporation, Tokyo, Japan). Podocyte apoptosis was defined as the double WT-1 and TUNEL staining of a cell, and double labeled/WT-1 staining ratio was calculated.

### Flow cytometry

MPC5 apoptosis was analyzed by flow cytometry using the PE Annexin V Apoptosis Detection kit (BD Biosciences). MPC5 cells were incubated in six-well plates. Following the treatment with NG, M, or HG for 48 h, or RNA transfection, the treated cells were collected, washed, and resuspended in 300 *μ*l of binding buffer, according to the manufacturer's instructions. The solution containing 3 *μ*l of PE Annexin and 3 *μ*l of 7-AAD was added, and the samples were incubated in the dark for 15 min at room temperature. These samples were analyzed using a cytometer (BD Biosciences).

### Immunofluorescence analysis

Mtdh and nephrin (podocyte marker) antibodies were used to investigate the location and expression of Mtdh in frozen renal tissue samples obtained from db/db and db/m mice. The slides were permeabilized with 0.05% Triton X-100 (Biosharp, Anhui, China) in PBS for 10 min and blocked with 5% goat serum mixed with 2.5% bovine serum albumin for 2 h. Afterward, these sections were incubated with anti-Mtdh antibody (1:100; Abcam) together with anti-nephrin antibody (1:50; Santa Cruz Biotechnology, Santa Cruz, CA, USA) at 4 °C overnight. Anti-Mtdh and anti-nephrin primary antibodies were detected using Alexa Fluor 546 donkey anti-rabbit and Alexa Fluor 488 goat anti-rabbit (1:1000; Invitrogen) secondary antibodies, respectively, and the samples were incubated with them for 6 h at 4 °C. Cell nuclei were stained with DAPI for 10 min before the observation of the samples under a light microscope (Nikon Corporation).

### Transient transfections with siRNAs, Mdth overexpression vector, miR-30 inhibitors, and miR-30 mimics

MPC5 were seeded at 2 × 10^5^ cells per well in six-well plates at 70% confluence after the differentiation. These cells were transfected with Mtdh siRNA (50 nM; GenePharma, Shanghai, China) or the overexpression (500 ng per well, GenePharma) the mixture of the mimics or inhibitors (RiboBio, Guangzhou, China) of all five miR-30 family members at the final concentrations of 50 nM, using Lipofectamine 2000 (Invitrogen) for 6 h in OPTI-MEM (Gibco BRL), according to the manufacturers' instructions. Afterward, OPTI-MEM was replaced with the complete medium containing 1% FBS, and treated with HG for 48 h after the transfection with siRNAs or mimics, whereas the cells treated with miR-30 inhibitors were not treated with HG. The sequences of Mtdh siRNAs were as follows: Mtdh 1: sense: 5′-GGAUGAAGUUGUUAGAAAUTT-3′, antisense: 5′-AUUUCUAACAACUUCAUCCTTUTT-3′ Mtdh 2: sense: 5′-GUCUACCACUUCUGAUUAUTTUTT-3′, antisense: 5′-AUAAUCAGAAGUGGUAGACTT-3′ Mtdh 3: sense: 5′-CGAGCCAUCUAUUACCUUATT-3′, antisense: 5′-UAAGGAAUAGAUGGCUCGTT-3′ and control: sense: 5′-UUCUCCGAACGUGUCACGUTT-3′, antisense: 5′-ACGUGACACGUUCGGAGAATT-3′.

### RNA extraction and qPCR

miRNAs and total RNA were extracted using TRIzol reagent (TransGen Biotech, Beijing, China), and qPCR was performed using a commercial kit (Takara Biotechnology, Dalian, China) according to the manufacturer's protocol. All experiments were performed in triplicate and the comparative assessment of target gene expression was performed using *β*-actin mRNA or U6 miRNA levels as internal controls. The primers used in this study are listed in Supplementary Information 1. Changes in levels analyzed by the relative quantification (ΔΔCt) method.

### Isolation of glomeruli

Glomeruli were isolated from streptozotocin-induced diabetic rats (approved by the ethics committee for the experimental use of animals at Southern Medical University, Guangzhou, China (L2015007) and kindly provided by Professor Hong-xin Niu form ZhuJiang Hospital, Southern Medical University, China) using the standard mechanical sieving technique, as previously described.^43^In brief, after mincing and grinding of the tissue, the renal cortex paste was forced through a series of mesh filters with 180-, 100-, 75-, and 70-*μ*m mesh openings. The tissue material remaining on the last sieve was rinsed and collected.

### Western blotting

Protein samples were prepared using radioimmunoprecipitation assay buffer, and protein concentrations were determined using the BCA kit (Thermo Fisher Scientific, Waltham, MA, USA). Equal amounts of proteins (50 *μ*g) were separated on the 12% sodium dodecyl sulfate-polyacrylamide gels, and transferred to polyvinylidene fluoride membranes (Millipore, Bedford, MA, USA). Following the blocking with 5% skimmed milk/TBST for 1 h, the membranes were incubated overnight at 4 °C with primary antibodies against Mtdh (1:800; Abcam), p38 MAPK, phosphorylated (p)-p38 MAPK, pro-caspase 3, and cleaved caspase 3 (1:1000; Cell Signaling Technologies, Danvers, MA, USA), *β*-actin (1:1000; EarthOx LLC, San Francisco, CA, USA), and Bax (1:800; Cell Signaling Technologies). The membranes were probed with the appropriate HRP-conjugated secondary antibodies (1:10 000; EarthOx) for 1 h at room temperature. Protein band intensities were quantified as described previously.^44^

### Dual-luciferase reporter assay

The 3′-UTR of Mtdh containing putative miR-30-binding sites was amplified and cloned into PmiR-RB-REPORT dual-luciferase reporter vector (RiboBio). In addition, a mutated Mtdh 3′-UTR was constructed (TGTTTAC (26–33, 284–291, 1547–1554) to ACAAATG; GTTTAC (563–569) to CAAATG). MPC5 cells in logarithmic growth phase were seeded into 96-well plates and incubated for 48 h before the transfection. miRNA mimics or NCs and luciferase reporter vector containing the 3′-UTR of Mtdh or the mutant sequence vector were co-transfected into the cells using Lipofectamine 2000 transfection reagent (Invitrogen) in OPTI-MEM (Gibco BRL). Cells were collected 48 h after the transfection and dual-luciferase reporter assay was performed (Promega, Madison, WI, USA). Firefly luciferase activity was normalized to the corresponding levels of renilla luciferase activity.

### Statistical analysis

Statistical analyses were performed with SPSS for Windows version 19.0 (SPSS, Chicago, IL, USA). All data were from at least three independent experiments. Data are presented as mean±S.E.M. Student's *t*-test was used for analyzing the differences between two groups, whereas ANOVA was used for the comparisons between multiple groups, followed by Student's *t*-test for the determination of differences between groups. *P*<0.05 was considered statistically significant in all cases.
