# Supplementary figures and images for: Metadherin facilitates podocyte apoptosis in diabetic nephropathy
Source: Cell Death Dis. 2016 Nov 24;7(11):e2477–. doi: 10.1038/cddis.2016.335 (PMC5260885; doi:10.1038/cddis.2016.335)

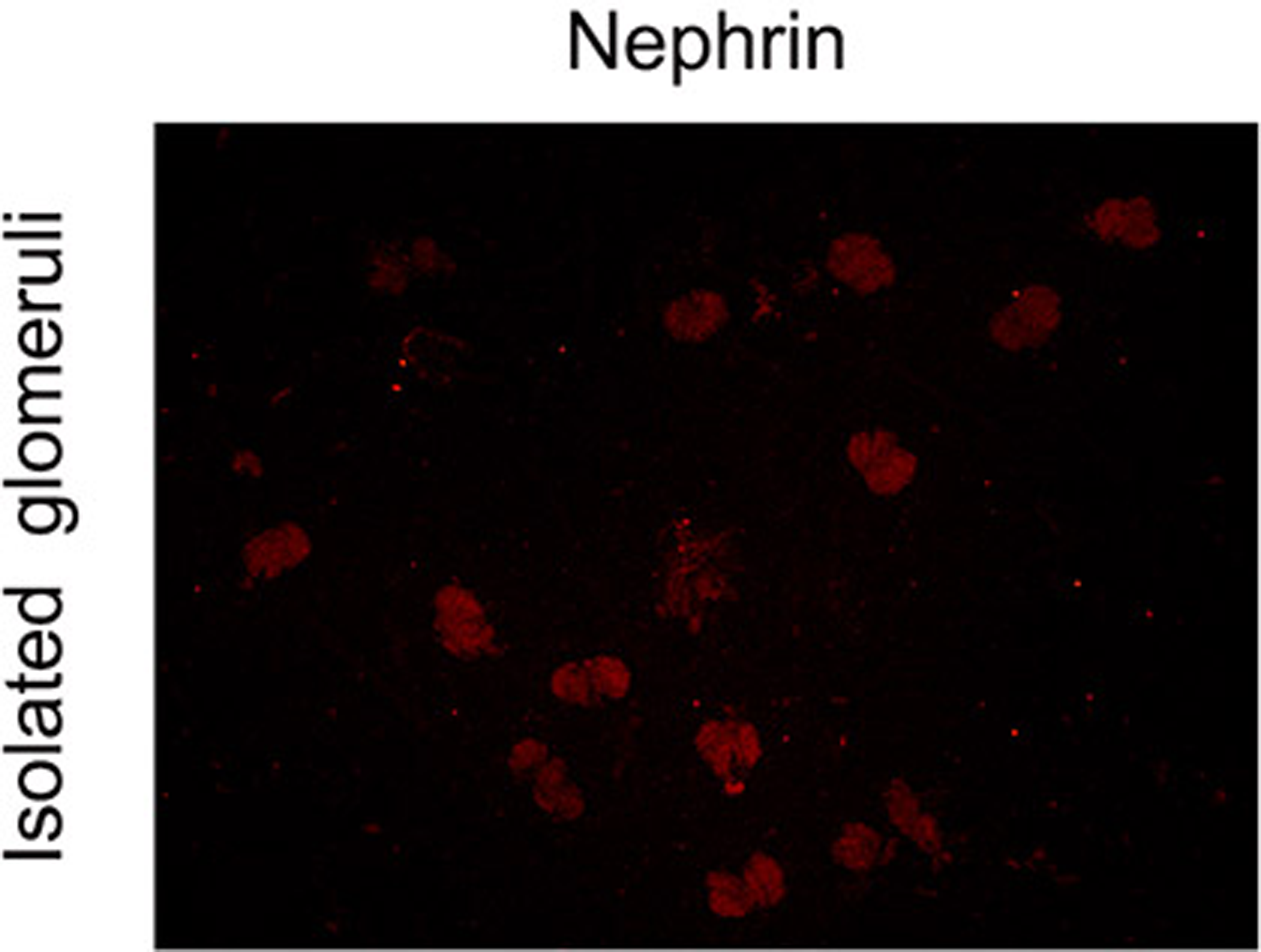

Supplement: Supplementary Figure 1 [file cddis2016335x1.tif]

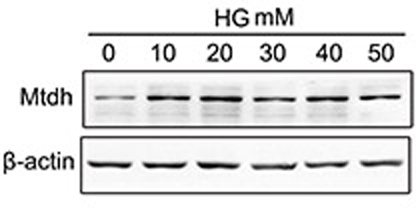

Supplement: Supplementary Figure 2 [file cddis2016335x2.tif]

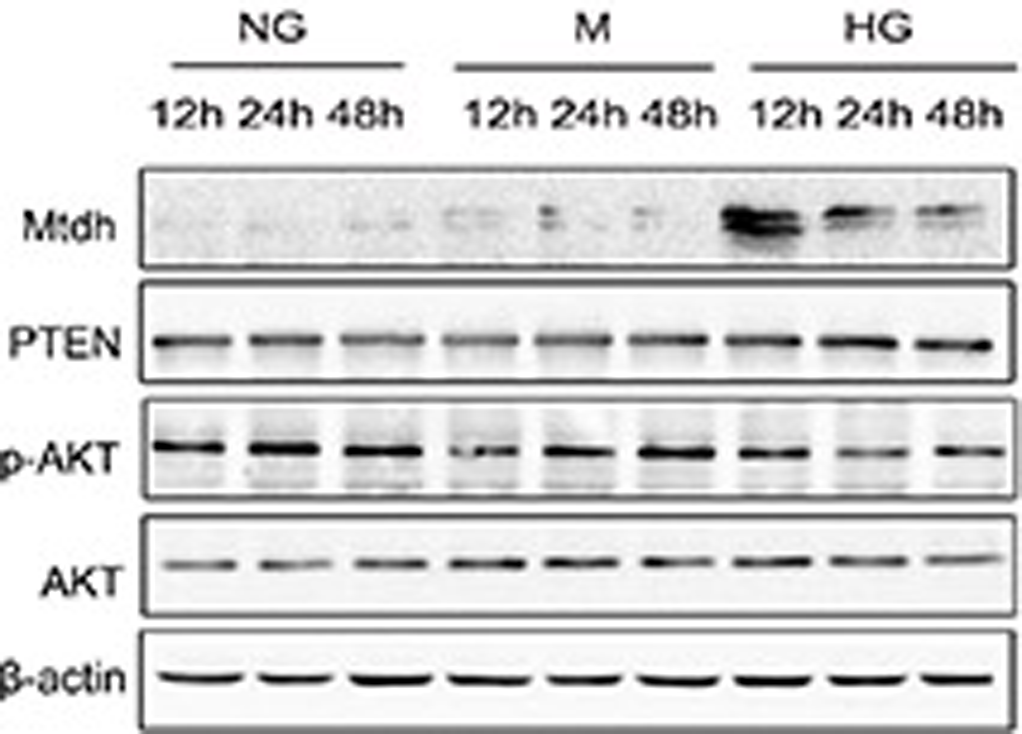

Supplement: Supplementary Figure 3 [file cddis2016335x3.tif]

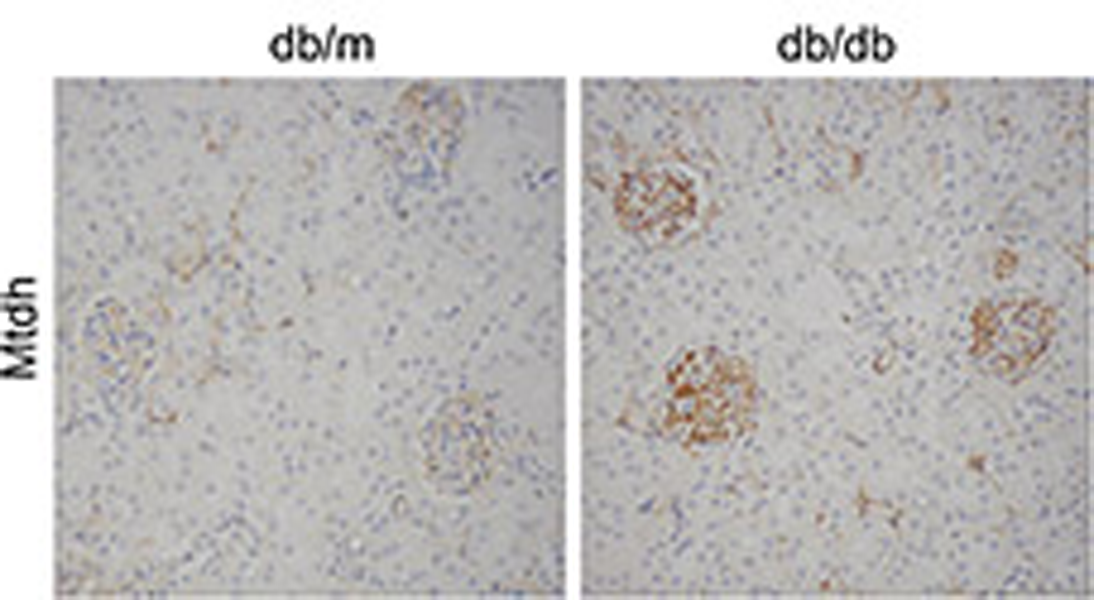

Supplement: Supplementary Figure 4 [file cddis2016335x4.tif]
