# Supplementary material for: Metadherin facilitates podocyte apoptosis in diabetic nephropathy
Source: Cell Death Dis. 2016 Nov 24;7(11):e2477–. doi: 10.1038/cddis.2016.335 (PMC5260885; doi:10.1038/cddis.2016.335)
Supplement: Supplementary Materials 1 [file cddis2016335x5.doc]

The primers for qPCR were as follows:

*Mtdh* forward(F): 5′-CTGCAAAACAAGCACCAGAG-3′,

reverse, (R):5′-AGTTTCCCAGGCTCCTTCAT-3′

β-actin F:5′-CACCTGCCATGTATGTAGC-3′,

R:5 ′-CATCCCCAGAATCCATCAC-3′.

| mmu-mir-30-a-RT | GTCGTATCCAGTGCGTGTCGTGGAGTCGGCAATTGCACTGGATACGACCTTCCAGT |
| --- | --- |
| mmu-mir-30-a-F | CGGTGTAAACATCCTCGACT |
| mmu-mir-30-a-R | CAGTGCGTGTCGTGGAGT |
| mmu-mir-30-b-RT | GTCGTATCCAGTGCGTGTCGTGGAGTCGGCAATTGCACTGGATACGACAGCTGAGT |
| mmu-mir-30-b-F | CACCCTGTAAACATCCTACACT |
| mmu-mir-30-b-R | CAGTGCGTGTCGTGGAGT |
| mmu-mir-30-1-c-RT | GTCGTATCCAGTGCGTGTCGTGGAGTCGGCAATTGCACTGGATACGACGCTGAGAG |
| mmu-mir-30-1-c-F | ACCCTGTAAACATCCTACACTC |
| mmu-mir-30-1-c-R | CAGTGCGTGTCGTGGAGT |
| mmu-mir-30-d-RT | GTCGTATCCAGTGCGTGTCGTGGAGTCGGCAATTGCACTGGATACGACCTTCCAGT |
| mmu-mir-30-d-F | CCCTGTAAACATCCCCGACT |
| mmu-mir-30-d-R | CAGTGCGTGTCGTGGAGT |
| mmu-mir-30-e-RT | GTCGTATCCAGTGCGTGTCGTGGAGTCGGCAATTGCACTGGATACGACCTTCCAGT |
| mmu-mir-30-e-F | CACCCTGTAAACATCCTTGACT |
| mmu-mir-30-e-R | CAGTGCGTGTCGTGGAGT |
