# Supplementary material for: Metadherin facilitates podocyte apoptosis in diabetic nephropathy
Source: Cell Death Dis. 2016 Nov 24;7(11):e2477–. doi: 10.1038/cddis.2016.335 (PMC5260885; doi:10.1038/cddis.2016.335)
Supplement: Supplementary Materials 2 [file cddis2016335x6.doc]

Figure 1:

Figure 1 e：Normal distribution test(Shapiro-Wilk): NG group :P=0.776; M group :P=0.509; HG group :0.516 ; Homogeneity of Variance test:p=0.073 ;n=4

Figure 1j：

12h WB Bax/β-actin Normal distribution test(Shapiro-Wilk): NG group :P=0.961; M group: P =0.237; HG group:0.948 ; Homogeneity of Variance test: p=0.181; n=3

24h WB Bax/β-actin Normal distribution test(Shapiro-Wilk): NG group: P=0.125; group M:P=0.0.957; HG group: p=0.718 ; Homogeneity of Variance test: p=0.077; n=3

48h WB Bax/β-actin Normal distribution test(Shapiro-Wilk): NG group :P=0.399; M group :P=0.105; HG group : p=0.638 ; Homogeneity of Variance test: p=0.136; n=3

Figure 1k :

12h cleaved caspase 3Normal distribution test(Shapiro-Wilk): NG group: P=0.107; M group: P=0.114; HG group: p=0.975 ; Homogeneity of Variance test: p=0.164; n=3

24h cleaved caspase 3 Normal distribution test(Shapiro-Wilk): NG group: P=0.735; M group: P=0.428; HG group: p=0.111; Homogeneity of Variance test: p=0.08; n=3

48h cleaved caspase 3 Normal distribution test(Shapiro-Wilk): NG group: P=0.243; M group: P=0.077; HG group: p=0.730 ; Homogeneity of Variance test: p=0.097; n=3

Figure 2:

Figure 2 d

Mtdh PCR 12h: Normal distribution test(Shapiro-Wilk): M group: P=0.991; HG group: p=0.470 .

Homogeneity of Variance test: p=0.051; n=4

Mtdh PCR 24h: Normal distribution test(Shapiro-Wilk): M group: P=1; HG group: p=0.298.

Homogeneity of Variance test: p=0.053; n=3

Mtdh PCR 48h: Normal distribution test(Shapiro-Wilk): M group: P=1; HG group: p=0.298.

Homogeneity of Variance test: p=0.106; n=3

Figure 2 e:

Mtdh 12 WB: Normal distribution test(Shapiro-Wilk): NG group: P=0.979; M group : p=0.051; HG group: p=0.656;Homogeneity of Variance test：p=0.089;n=4

Mtdh 24 WB: Normal distribution test(Shapiro-Wilk): NG group :P=0.278; M group : p=0.213; HG group: p=0.153; Homogeneity of Variance test: p=0.066;n=3

Mtdh 48 WB: Normal distribution test(Shapiro-Wilk): NG group: P=0.962; M group : p=0.457; HG group: p=0.843; Homogeneity of Variance test: p=0.059;n=3

Figure 3:

Figure 3 a：Normal distribution test(Shapiro-Wilk): NG group: P=0.339; HG group: p=0.677

Homogeneity of Variance test: p=0.140;n=3

Figure 3 b：Normal distribution test(Shapiro-Wilk): NG group: P=0.866; HG group: p=0.679

Homogeneity of Variance test: p=0.299;n=3

Figure 3 c：Normal distribution test(Shapiro-Wilk): NG group: P=0.866; HG group: p=0.679

Homogeneity of Variance test: p=0.299;n=3

Figure 3 d：Normal distribution test(Shapiro-Wilk): NG group: P=0.862; HG group: p=0.900

Homogeneity of Variance test: p=0.661;n=3

Figure 3 e：Normal distribution test(Shapiro-Wilk): NG group: P=0.482; HG group: p=0.194

Homogeneity of Variance test: p=0.125;n=3

Figure 3f: Normal distribution test(Shapiro-Wilk): NG group: P=0.174; HG group: p=0.313

Homogeneity of Variance test: p=0.348;n=4

Figure 3h: Nrmal distribution test(Shapiro-Wilk): NC group: P=0.943; Mtdh plasimids group: p=0.619 ;Homogeneity of Variance test: p=0.067;n=3

Figure 3J：Normal distribution test(Shapiro-Wilk): NC group: P=0.938; Mtdh plasimids group: p=0.194 ;Homogeneity of Variance test: p=0.148;n=3

Figure 3K：Normal distribution test(Shapiro-Wilk): NC group: P=0.248; Mtdh plasimids group: p=0.831 ;Homogeneity of Variance test: p=0.346;n=3

Figure 4:

Figure 4 d:

12h: Normal distribution test(Shapiro-Wilk): NG group: P=0.134; M group: p=0.646; HG group: p=0.271;Homogeneity of Variance test: p=0.151;n=3

24h:Normal distribution test(Shapiro-Wilk): NG group :P=0.123; M group: p=0.522; HG group: p=0.381; Homogeneity of Variance test: p=0.140;n=3

48h:Normal distribution test(Shapiro-Wilk): NG group: P=0.911; M group: p=0.842; HG group: p=0.319; Homogeneity of Variance test: p=0.503;n=3

Figure 4 f: p-p38/p38 Normal distribution test(Shapiro-Wilk): NG group: P=0.755; SB(-)+ HG group: p=0.190; SB(+)+ HG group: p=0.135; Homogeneity of Variance test: p=0.656; n=3

Figure 4 g: BAX/Β-ACTIN Normal distribution test(Shapiro-Wilk): NG group: P=0.443; SB(-)+ HG group: p=0.806; SB(+)+ HG group: p=0.742; Homogeneity of Variance test: p=0.911; n=3

Figure 4 h:cleaved capase3/pro-caspase 3 Normal distribution test(Shapiro-Wilk): NG group: P=0.213; SB(-)+ HG group: p=0.413; SB(+)+ HG group: p=0.274; Homogeneity of Variance test: p=0.062; n=3

Figure 4 I : p-p38/p38 Normal distribution test(Shapiro-Wilk): HG+NC group: P=0.407; HG +si-Mtdh group: p=0.132; Homogeneity of Variance test: p=0.356; n=3

Figure 4 j : p-p38/p38 Normal distribution test(Shapiro-Wilk): HG+NC group: P=0.460; HG +si-Mtdh group: p=0.158; Homogeneity of Variance test: p=0.050; n=3

Figure 5:

Figure 5 **b**

MiR-30a : Normal distribution test(Shapiro-Wilk)：p=0.983，Homogeneity of Variance test: p=0.112; n=3

MiR-30b : Normal distribution test(Shapiro-Wilk)：p=0.817，Homogeneity of Variance test: p=0.078; n=3

MiR-30c : Normal distribution test(Shapiro-Wilk)：p=0.662，Homogeneity of Variance test: p=0.053; n=3

MiR-30d : Normal distribution test(Shapiro-Wilk)：p=0.698，Homogeneity of Variance test: p=0.058; n=3

MiR-30e : Normal distribution test(Shapiro-Wilk)：p=0.259，Homogeneity of Variance test: p=0.021; n=3

Figure 5 c

MiR-30a 12h group: Normal distribution test(Shapiro-Wilk)：p=0.256，Homogeneity of Variance test: p=0.050; n=3

MiR-30a 24h group: Normal distribution test(Shapiro-Wilk)：p=0.345，Homogeneity of Variance test: p=0.052; n=3

MiR-30a 48h group: Normal distribution test(Shapiro-Wilk)：p=0.086，Homogeneity of Variance test; p=0.086; n=3

MiR-30b 12h group: Normal distribution test(Shapiro-Wilk)：p=0.250，Homogeneity of Variance test: p=0.052; n=3

MiR-30b 24h group: Normal distribution test(Shapiro-Wilk)：p=0.328，Homogeneity of Variance test: p=0.052; n=3

MiR-30b 48h group: Normal distribution test(Shapiro-Wilk)：p=0.565，Homogeneity of Variance test: p=0.054; n=3

MiR-30c 12h group: Normal distribution test(Shapiro-Wilk)：p=0.565，Homogeneity of Variance test: p=0.052; n=3

MiR-30c 24h group: Normal distribution test(Shapiro-Wilk)：p=0.052，Homogeneity of Variance test: p= 0.052; n=3

MiR-30c 48h group: Normal distribution test(Shapiro-Wilk)：p=0.415，Homogeneity of Variance test: p=0.054; n=3

MiR-30d 12h group: Normal distribution test(Shapiro-Wilk)：p=0.474，Homogeneity of Variance test: p=0.053; n=3

MiR-30d 24h: Normal distribution test(Shapiro-Wilk)：p=0.215，Homogeneity of Variance test: p= 0.032; n=3

MiR-30d 48h group: Normal distribution test(Shapiro-Wilk)：p=0.812，Homogeneity of Variance test: p=0.077; n=3

MiR-30e 12h group: Normal distribution test(Shapiro-Wilk)：p=0.057，Homogeneity of Variance test: p=0.050; n=3

MiR-30e 24h group: Normal distribution test(Shapiro-Wilk)：p=1.00，Homogeneity of Variance test: p= 0.116; n=3

MiR-30e 48h group: Normal distribution test(Shapiro-Wilk)：p=0.195，Homogeneity of Variance test: p=0.052; n=3

Figure 5 d: miR-30a

Normal distribution test(Shapiro-Wilk):Mtdh WT group: p= 0.712; Mtdh WT+ mmu group: p= 0.328; Homogeneity of Variance test: p=0.693; n=3

Normal distribution test(Shapiro-Wilk):Mtdh Mut group: p= 0.900; Mtdh Mut+ mmu group: p= 0.433;Homogeneity of Variance test: p=0.922; n=3

Figure 5 e: miR-30b

Normal distribution test(Shapiro-Wilk):Mtdh WT group: p= 0.747; Mtdh WT+ mmu group: p= 0.848;Homogeneity of Variance test: p=0.192; n=3

Normal distribution test(Shapiro-Wilk):Mtdh Mut group: p= 0.433; Mtdh Mut+ mmu group: p= 0.588; Homogeneity of Variance test: p=0.379; n=3

Figure 5 f: miR-30c

Normal distribution test(Shapiro-Wilk):Mtdh WT group: p= 0.780; Mtdh WT+ mmu group: p= 0.463; Homogeneity of Variance test: p=0.776; n=3

Normal distribution test(Shapiro-Wilk):Mtdh Mut group: p= 0.747 ;Mtdh Mut+ mmu group: p= 0.545;Homogeneity of Variance test: p=0.200; n=3

Figure 5 g: miR-30d

Normal distribution test(Shapiro-Wilk):Mtdh WT group: p= 0.868; Mtdh WT+ mmu group: p= 0.463;Homogeneity of Variance test: p=0.234; n=3

Normal distribution test(Shapiro-Wilk):Mtdh Mut group: p= 0.463; Mtdh Mut+ mmu group: p= 0.900;Homogeneity of Variance test: p=0.753; n=3

Figure 5 h :miR-30e

Normal distribution test(Shapiro-Wilk):Mtdh WT group: p= 0.702; Mtdh WT+ mmu group: p= 0.637;Homogeneity of Variance test: p=0.195; n=3

Normal distribution test(Shapiro-Wilk):Mtdh Mut group: p= 0.1; Mtdh Mut+ mmu group p= 0.222;Homogeneity of Variance test: p=0.089; n=3

Figure 6

Figure 6 a：

25nM miR-30a inhibitor: Normal distribution test(Shapiro-Wilk)：p=0.771; Homogeneity of Variance test：p=0.070; n=3

50nM miR-30a inhibitor: Normal distribution test(Shapiro-Wilk)：p=0.917; Homogeneity of Variance test：p=0.097; n=3

100nM miR-30a inhibitor: Normal distribution test(Shapiro-Wilk)：p=0.902; Homogeneity of Variance test：p=0.094; n=3

Figure 6 b：

25nM miR-30a mimic: Normal distribution test(Shapiro-Wilk)：p=0.643; Homogeneity of Variance test：p=0.051; n=3

50nM miR-30a mimic: Normal distribution test(Shapiro-Wilk)：p=0.485; Homogeneity of Variance test：p=0.034; n=3

100nM miR-30a mimic: Normal distribution test(Shapiro-Wilk)：p=0.833; Homogeneity of Variance test：p=0.080; n=3

Figure 6 c：Normal distribution test(Shapiro-Wilk)：p=0.745; Homogeneity of Variance test：p=0.065; n=3

Figure 6 d：Normal distribution test(Shapiro-Wilk)：p=0.373; Homogeneity of Variance test：p=0.052; n=3

Figure6 e：Normal distribution test(Shapiro-Wilk)：HG+NC group :p=0.180; HG+si-Mtdh group p:=0.180;Homogeneity of Variance test：p=0.579; n=4

Figure 6 f：Normal distribution test(Shapiro-Wilk)：HG+NC group :p=0.546; HG+si-Mtdh group p:=0.341; Homogeneity of Variance test：p=0.226; n=3

Figure 7

Figure7a：Normal distribution test(Shapiro-Wilk)：NC group: p=0.581; miR-30s inhibitor group p=0.181;Homogeneity of Variance test：p=0.511 n=3

Figure 7 b：Normal distribution test(Shapiro-Wilk)：HG+NC group: p=0.581; HG+miR-30s mimics group p=0.181; Homogeneity of Variance test：p=0.052 ;n=3

Figure 7 c：

Normal distribution test(Shapiro-Wilk)：Bax/β-actin/B-actin: NC group: p=0.756; miR-30s inhibitor group: p=0.548;Homogeneity of Variance test：p=0.070; n=3

Normal distribution test(Shapiro-Wilk)：cleaved caspase 3/pro-caspase 3: NC group: p=0.240; miR-30s inhibitor group: p=0.851Homogeneity of Variance test：p=0.051 ;n=4

Figure 7 d：

Normal distribution test(Shapiro-Wilk)：Bax/β-actin/B-actin :HG+NC group: p=0.855; HG+miR-30s mimics group: p=0.919; Homogeneity of Variance test：p=0.236 ；n=3

Normal distribution test(Shapiro-Wilk)：cleaved caspase 3/pro-caspase 3:HG+NC group: p=0.886; HG+miR-30s mimics group: p=0.991; Homogeneity of Variance test：p=0.822； n=3
